# Temporal variations in health risk indices and combustion-derived components of PM_1.0_: Focus on terephthalate and levoglucosan

**DOI:** 10.1016/j.heliyon.2024.e40052

**Published:** 2024-11-09

**Authors:** Myoungki Song, Seoyeong Choe, Sea-Ho Oh, Minyoung Sung, Ji Yun Jung, Jinsoo Choi, Joonyoung Ahn, Jungmin Park, Myungsoo Yoo, Jinsoo Park, Min-Suk Bae

**Affiliations:** a Department of Environmental Engineering, Mokpo National University, Muan, Republic of Korea; b Climate and Air Quality Research Department, Air Quality Research Division, National Institute of Environmental Research, Incheon, Republic of Korea; c Climate and Air Quality Research Department, National Institute of Environmental Research, Incheon, Republic of Korea

**Keywords:** Oxidative potential, Risk assessment, Terephthalic acid, Polycyclic aromatic hydrocarbons, Levoglucosan

## Abstract

This study evaluated the health risks and chemical composition of PM_1.0_ and PM_2.5_ in Incheon, South Korea, emphasizing the critical role of particle size in public health impacts. The average concentrations were 10.89 μg/m³ for PM_2.5_ and 8.11 μg/m³ for PM_1.0_. PM_1.0_ displayed higher proportions of carbonaceous components and water-soluble ions, predominantly formed through photochemical reactions and atmospheric chemistry processes. Health risk assessments, using the Benzo [a]pyrene toxic equivalency factor, mutagenic and carcinogenic potential, risk index, and oxidative potential (DTT-OP), indicated that PM_1.0_ poses significantly higher health risks per unit mass compared to PM_2.5_. Key components in PM_1.0_, such as levoglucosan and terephthalic acid (TPA), indicate significant contributions from combustion sources like biomass burning and plastic incineration, particularly at night. PM_1.0_ showed higher carcinogenic and mutagenic risks than PM_2.5_. The correlation between levoglucosan, PAHs, and TPA supports common combustion origins. Effective management of combustion-related emissions is crucial for reducing health risks associated with PM_1.0_. The overall average risk index for PM_1.0_ is 1.30 times higher than PM_2.5_, implying that, on average, PM_1.0_ poses a 30 % higher health risk across the measured indices compared to PM_2.5_. This study emphasizes the need for targeted management of combustion emissions, particularly those from plastic and fuel combustion, to mitigate the health risks posed by PM_1.0_. Effective control of the precursors contributing to PM_1.0_ formation is crucial for reducing the adverse health impacts of air pollution.

## Highlights

• PM_1.0_ contains higher proportions of carbonaceous components than PM_2.5_.
• PM_1.0_ has a greater carcinogenic and mutagenic risk than PM_2.5_.
• Nighttime combustion increases levoglucosan and terephthalate levels in PM_1.0_.

## Abbreviations

17α(H),21β(H)-30-Norhopane: C29αβ
17α(H),21β(H)-Hopane: C30αβ
Acenaphthene: Ace
Acenaphthylene: Acy
Anthracene: Ant
Benzo[a]anthracane: BaA
Benzo[a]pyrene: BaP
Benzo[b]fluoranthene: BbF
Benzo[e]pyrene: BeP
Benzo[g,h,i]perylene: BghiP
Benzo[k]fluoranthene: BkF
Chrysene: Chry
Cyclopenta[cd]pyrene: CcdP
Dibenz[a,h]anthracane: DahA
Dithiothreitol: DTT
Dithiothreitol-oxidative potential: DTT-OP
Dithiothreitol-oxidative potential normalized to 9,10-phenanthrenequinone: (QDTT-OP)
Fluoranthene: Flt
Fluorene: Flu
Indeno123cdpyrene: IndP
methanesulfonic acid: MSA
mutagenic potency factor of PAHs relative to BaP: MEF
Naphtalene: NaP
Phenanthrene: Phen
phthalic acid: PA
Polyethylene terephthalate: PET
potency equivalency factor of PAHs relative to BaP: PEF
Pyrene: Pyr
Reactive oxygen species: ROS
terephthalic acid: TPA
Toxic equivalency factors for carcinogenicity relative to BaP: TEF
Water insoluble OC: WIOC
Water soluble OC: WSOC

## 1 Background

Particulate matter (PM) represents one of the most significant public health threats globally [[1], [2], [3], [4]]. As of 2019, ambient particulate matter was identified as a foremost contributor to global disability-adjusted life years (DALYs) and was recognized by the World Health Organization (WHO) as a primary cause of death from cardiovascular and respiratory diseases [5,6]. A crucial factor influencing the impact of particulate matter on human health is the size of the particles [7]. Smaller particles are generally understood to have a more significant effect on human health. Research has shown that particles with diameters between 2 and 10 μm can be inhaled into the human body, with approximately 10 % of these particles reaching the lungs. Importantly, particles with diameters from 0.3 to 2 μm almost entirely penetrate the alveoli [8]. Consequently, smaller particles enhance toxicity through mechanisms like oxidative stress and inflammation within the human body [9]. Smaller particle sizes increase the specific surface area per unit weight, facilitating the adsorption of various air pollutants on the particle surface [10]. For instance, the porous structures on the surface of particulate matter have been demonstrated to absorb and adsorb deleterious substances like polycyclic aromatic hydrocarbons (PAHs) [11]. Thus, the smaller the particulate matter, the greater the potential for particle-induced toxicity and the transport of various hazardous substances to the alveoli, leading to considerable health risks.

With the known risks associated with the size of particulate matter, research has increasingly focused on smaller particles, such as PM_10_ and PM_2.5_. Most contemporary studies have targeted PM_2.5_, revealing various insights concerning its chemical composition, sources, and associated health risks [[12], [13], [14]]. However, given that smaller particles pose greater health risks, attention to particles smaller than PM_2.5_ is emerging. Recent investigations have shown that PM_1.0_, with an aerodynamic diameter of less than 1 μm, penetrates deeper into the respiratory system and reaches the lungs more readily than PM_2.5_, sparking increased interest in PM_1.0_ [15]. However, the research on PM_1.0_ still shows inconsistent conclusions, and the study of its physicochemical characteristics and sources remains limited. PM_1.0_, posing significant human health risks, originates predominantly from secondary aerosol formation through photochemical reactions of gaseous substances and atmospheric chemical reactions involving volatile organic compounds (VOCs) and organic carbon [13,16]. Precursor substances involved in these secondary aerosols and their emission sources strongly influence the health risks associated with PM_1.0_ [[16], [17], [18]]. Notably, health risk assessments of particulate matter, based on ROS levels, indicate higher risks from combustion sources such as biomass burning compared to other sources [[16], [17], [18], [19]]. In urban areas, PM_2.5_, known for its high health risks, contains terephthalic acid (TPA) and levoglucosan [17]. TPA is typically recognized as a secondary water-soluble organic acid indicator. Recent studies, however, have identified TPA as a primary combustion marker for plastics, generated alongside ethylene during the thermal decomposition of polyethylene terephthalic acid (PET) [17,[20], [21], [22]]. Similarly, levoglucosan is formed during combustion processes, linking it to secondary aerosol formation from PET combustion. Consequently, PM_2.5_ derived from TPA is presumed to pose substantial health risks due to its PM_1.0_ size and association with combustion sources.

This study aims to analyze the chemical composition of PM_1.0_ compared to PM_2.5_, elucidating characteristics distinctive to PM_1.0_. Additionally, it explores the health risks posed by PM_1.0_ based on chemical analysis results and aims to identify the sources of hazardous substances within PM_1.0_. PM_1.0_ and PM_2.5_ were simultaneously collected and chemically analyzed in this research, providing foundational data expected to inform future studies on the significant health risks associated with PM_1.0_.

## 2 Research method

### 2.1 Measurement location

**Fig. 1:**
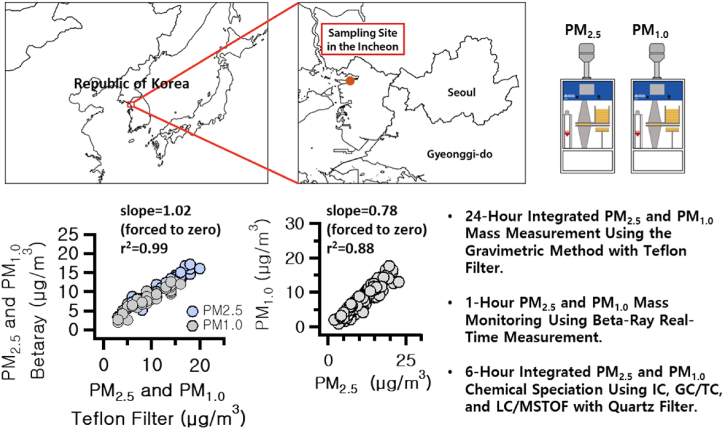
Overview of the study area, comparative analysis of PM_2.5_ and PM_1.0_ mass concentrations using beta-ray and gravimetric methods, and evaluation of PM_1.0_ mass concentrations via the beta-ray method.

The measurement of PM_2.5_ and PM_1.0_ in the ambient air took place in Incheon, South Korea (latitude: 34.3412, longitude: 126.3825) (Fig. 1). Sampling occurred from 00:00 on August 17, 2023, to 18:00 on September 26, 2023, with collections made every 6 h, yielding 4 p.m._2.5_ and PM_1.0_ samples daily. The National Institute of Environmental Research (NIER) in South Korea, free of residential and industrial developments within a 1 km radius, served as the sampling site. While an industrial complex lies 4.5 km northwest of the site, the prevailing parkland setting likely minimizes the industrial influence. The detailed sampling location can be found from the previous studies [23,24].

### 2.2 Chemical analysis of PM _1.0_ and PM _2.5_

The observation parameters were classified into two categories: the concentrations of PM_2.5_ and PM_1.0_, and their component analyses. The concentrations were determined using a Beta Ray Attenuation Monitor with an impactor cut (BAM-1022, Met One Instruments Inc., USA). For quality assurance, separate measurements obtained through Teflon filters were compared with results from 24-h Beta Ray assessments. As depicted in Fig. 1, the reliability of the Beta Ray results was substantiated. Additionally, component analysis of PM_2.5_ and PM_1.0_ was conducted using samples collected on quartz filters, which were pre-baked at 450 °C for at least 6 h, utilizing a High Volume Aerosol Sampler (DHA-80, Digitel Elektronik AG, Switzerland). The analyzed components included organic carbon and elemental carbon (OCEC), water-soluble ions (NO_3_^−^, SO_4_^2−^, NH_4_^+^), 22 PAHs compounds including BaP, seven hopanes and steranes, and polar organic compounds such as PA, MSA, TPA, and levoglucosan. Analysis of OCEC was performed with a Carbon Aerosol Analyzer (Lab -based OCEC Carbon Aerosol Analyzer, Sunset Laboratory Inc., USA) following the National Institute of Occupational Safety & Health (NIOSH 5040) protocol. Quantitative analysis of water-soluble ion components commenced post-extraction from cut sample filters using 10 mL of distilled water, facilitated by an ultrasonic cleaner (8800, Branson, USA) connected to a thermostatic fluid circulator (CA-111, Eyela, Japan) for 2 h. Anions and cations were analyzed using ion chromatography (Metrohm 930, Switzerland) with a Metrosep A Supp 150/4.0 column (3.7 mM Na_2_CO_3_ & 1.0 mM NaHCO_3_) for anions, and a Metrosep C4-250/4.0 column (5 mM HNO_3_) for cations.

For the analysis of PAHs, hopanes, and steranes contained in PM_2.5_ and PM_1.0_, the collected filters were injected with the internal standard substance Pyrene-d_10_ as the internal standard, then subjected to solvent extraction and concentration using an Extreva ASE extraction evaporation system (Thermo, USA) with a dichloromethane:hexane (9:1) solvent mix. The concentrated extracts were subsequently analyzed by Gas Chromatography-Tandem Mass Spectrometry (GC-TM) (Agilent 8890 GC, 7010B GC/TQ) in multiple reaction monitoring (MRM) mode. Organic components, including PA, TPA, MSA, and levoglucosan, were analyzed by ultrasonically extracting sample filters using a 1:1 water/methanol solution and subsequently analyzed using an LC-QToF setup (LC: Agilent Infinity II, QToF: SCIEX X500r) equipped with an SB-C18 column (Agilent Poroshell 120). The comprehensive Quality Assurance/Quality Control (QA/QC) procedures can be reviewed in prior studies [13,16,17,25,26].

### 2.3 Human risk assessment of PM_1.0_ and PM_2.5_

The health risk assessment for PM_1.0_ and PM_2.5_ utilized the analyzed results of PAHs and the QDTT-OP method to evaluate ROS, which are recognized as inducers of inflammation. The health risks associated with PAHs were classified into categories of carcinogenicity, mutagenic potency, and non-carcinogenic risks. Specifically, carcinogenicity was quantified using BaP_TEQ_, based on the toxic equivalency factor (TEF) of BaP. Mutagenic potency was expressed as BaP_MEQ_, similarly employing the TEF. BaP_TEQ_ was calculated using two methods: the Nibet and LaGoy method, which considers 16 types of PAHs, and the USEPA (1993) method, encompassing 7 types of PAHs. The assessment of carcinogenicity using BaP_TEQ_ involved evaluating the relative toxicity of various PAHs compared to BaP, the most toxic among them. Additionally, to appraise the carcinogenicity of PAH mixtures, BaP_PEQ_ was computed, estimating the carcinogenic potential of specific PAHs relative to BaP based on potency equivalency factors (PEF) as opposed to TEFs [[27], [28], [29], [30], [31], [32]].

### 2.4 Analytical method for dithiothreitol assay-oxidative potential normalized to 9,10-phenanthrenequinone (QDTT-OP)

The QDTT-OP analysis represents an enhancement of the DTT-OP assay, in which the reduction of DTT is normalized using quinone. This reaction integrates the redox-active components of PM_2.5_, specifically targeting the DTT depletion rate influenced by various chemical components, including ROS found in PM_2.5_ [33,34]. However, the DTT depletion rate in the traditional DTT-OP assay is affected by the initial DTT concentration used in the assay, which complicates quantitative assessments of human health risks associated with the DTT-OP method [35]. The QDTT-OP method efficiently addresses this limitation by normalizing the DTT depletion through the concentration of 9,10-Phenanthrenequinone and subsequently assessing the oxidative potential of PM_2.5_ samples [16]. The analysis begins by preparing a solution extracted from PM_2.5_ and PM_1.0_, adjusting the pH to 7.4 through the addition of 0.2 mM DTT, 2.41 mM 5,5-dithio-bis (2-nitrobenzoic acid) (DTNB), and 500 mM potassium phosphate monobasic, followed by adding 100 mM potassium phosphate dibasic. The reaction mixture undergoes homogenization using a Multiflo FX Multi-Mode Dispenser (Agilent Technologies, USA), and is then incubated at 37 °C. Absorbance of TNB at 412 nm is measured four times across 40 min. For ensuring analytical precision within 5 %, the assays are replicated after every 15 samples and validated by introducing quinone samples. Detailed Quality Assurance/Quality Control (QA/QC) protocols are outlined in previous studies [13,14,17,36,37].

## 3 Results

### 3.1 Characteristics of PM_2.5_ and PM_1.0_ in the study area

During the study period, the average concentrations of PM_2.5_ and PM_1.0_ were 10.89 μg/m³ and 8.11 μg/m³, respectively (Table 1). The correlation coefficient of determination (r^2^) for the daily time series variations of PM_2.5_ and PM_1.0_ was 0.88, demonstrating a consistent pattern of variation between the two, with PM_1.0_ constituting approximately 74 % of PM_2.5_. The composition of PM_2.5_ included roughly 38.29 % carbonaceous components (OC and EC) and 39.21 % water-soluble ions (NH_4_^+^, SO_4_^2−^, NO_3_^−^), making up 77.50 % of the total PM_2.5_. In comparison, PM_1.0_ had higher proportions of carbonaceous components (44.64 %) and water-soluble ions (47.72 %). From the component analysis of PM_2.5_ and PM_1.0_, the characteristics of particulate matter in the study area were identified. Typically, WSOC and water-soluble ions within OC are indicative of secondary aerosol materials that form through photochemical reactions or aqueous chemistry involving strong solar radiation, high temperatures, and high O_3_ concentrations [[38], [39], [40], [41], [42]]. Analysis of the differences in carbonaceous and water-soluble ion components between PM_2.5_ and PM_1.0_ provides insight into the formation characteristics of these particulates. The OC/EC ratio for PM_2.5_ was 11.26, with an OC concentration of 3.83 μg/m³ and an EC concentration of 0.34 μg/m³. For PM_1.0_, the OC/EC ratio was 13.48, with an OC concentration of 3.37 μg/m³ and an EC concentration of 0.25 μg/m³. The median OC/EC ratios were 12.14 for PM_2.5_ and 14.74 for PM_1.0_, achieving their peaks from 12:00 to 18:00 (PM_2.5_: 16.40, PM_1.0_: 24.78).

**Table 1:** Summary statistics of average concentrations of measured species in study area.

Compounds	Unit	PM_2.5_	PM_1.0_	PM_1.0_/PM_2.5_^e^
**PM**_**2.5**_**& PM**_**1.0**_**mass**	μg/m³	10.89 ± 4.38	8.11 ± 3.7	0.74
**QDDT-OP**^a^	μM/m³	0.34 ± 0.11	0.31 ± 0.12	0.91
**OC**	μg/m³	3.83 ± 1.49	3.37 ± 1.29	0.88
**EC**	μg/m³	0.34 ± 0.23	0.25 ± 0.20	0.74
**NO**_**3**_^**−**^	μg/m³	0.58 ± 0.96	0.57 ± 0.82	0.98
**SO**_**4**_^**2-**^	μg/m³	2.62 ± 1.82	2.30 ± 1.66	0.88
**NH**_**4**_^**+**^	μg/m³	1.07 ± 0.78	1.00 ± 0.68	0.94
**Levoglucosan**	ng/m³	43.53 ± 44.68	32.27 ± 34.8	0.74
**PA**^b^	ng/m³	5.48 ± 3.77	4.42 ± 3.01	0.81
**TPA**^c^	ng/m³	19.67 ± 23.83	14.88 ± 17.03	0.76
**MSA**^d^	ng/m³	24.94 ± 15.90	21.95 ± 15.76	0.88
**Phenanthrene**	ng/m³	0.079 ± 0.028	0.028 ± 0.072	0.91
**Anthracene**	ng/m³	0.078 ± 0.025	0.025 ± 0.073	0.94
**9-Methylanthracene**	ng/m³	0.007 ± 0.005	0.005 ± 0.006	0.86
**Fluoranthene**	ng/m³	0.158 ± 0.066	0.066 ± 0.129	0.82
**Pyrene**	ng/m³	0.330 ± 0.327	0.327 ± 0.240	0.73
**Hexadecylcyclohexane**	ng/m³	0.047 ± 0.018	0.018 ± 0.033	0.86
**Heptadecylcyclohexane**	ng/m³	0.098 ± 0.045	0.045 ± 0.043	0.44
**Benzo[ghi]flouranthene**	ng/m³	0.022 ± 0.019	0.019 ± 0.018	0.85
**Cyclopenta[cd]pyrene**	ng/m³	0.110 ± 0.053	0.053 ± 0.071	0.64
**Benzo[a]anthracene**	ng/m³	0.090 ± 0.053	0.053 ± 0.086	0.96
**Chrysene**	ng/m³	0.138 ± 0.055	0.055 ± 0.137	0.99
**Benzo[a]pyrene**	ng/m³	0.247 ± 0.188	0.188 ± 0.12	0.49
**Perylene**	ng/m³	0.317 ± 0.304	0.304 ± 0.099	0.31
**Dibenzo[a,h]anthracene**	ng/m³	0.113 ± 0.124	0.124 ± 0.089	0.79
**Benzo[ghi]perylene**	ng/m³	0.181 ± 0.149	0.149 ± 0.144	0.82
**ααα-20S-C27-Cholestane**	ng/m³	0.025 ± 0.045	0.018 ± 0.018	0.72
**αββ-20R-C28-Methylcholestane**	ng/m³	0.017 ± 0.013	0.010 ± 0.010	0.59
**αββ-20R-C29-Ethylcholestane**	ng/m³	0.018 ± 0.024	0.012 ± 0.012	0.67
**17α (H),22,29,30-Trisnorhopane**	ng/m³	0.298 ± 0.548	0.181 ± 0.181	0.61
**17α (H),21β (H)-30-Norhopane**	ng/m³	0.144 ± 0.309	0.053 ± 0.053	0.37
**17α (H),21β (H)-Hopane**	ng/m³	0.402 ± 0.882	0.044 ± 0.044	0.11
**17α (H),21β (H)-22S-Homohopane**	ng/m³	0.510 ± 1.027	0.102 ± 0.102	0.20

a dithiotheitol assay-oxidative potential normalized to 9,10-phenanthrenequinone.

b phthalicacid.

c telephthalic acid.

d methylsufonic acid.

e unitless.

Furthermore, the proportion of PA, a water-soluble organic acid resulting from atmospheric chemical reactions, was notably higher in PM_1.0_ compared to PM_2.5_, with peak levels from 12:00 to 18:00 [19]. Additionally, the r^2^ values for the correlation between PA and OC were 0.49 for PM_2.5_ and 0.52 for PM_1.0_, suggesting a stronger influence of PA in PM_1.0_. These findings indicate that a significant portion of OC in the particulates is formed through photochemical reactions, with the resulting particles predominantly being of PM_1.0_ size. The characteristics of water-soluble ions in PM_2.5_ and PM_1.0_ were also investigated._1.0_ were also investigated. The proportion of water-soluble ions constituted 39.21 % of PM_2.5_ and 47.72 % of PM_1.0_. When assessed based on atmospheric concentrations, the levels of NO_3_^−^ were measured at 0.58 μg/m³ in PM_2.5_ and 0.57 μg/m³ in PM_1.0_, while the levels of SO_4_^2−^ were 2.62 μg/m³ and 2.30 μg/m³ in PM_2.5_ and PM_1.0_, respectively. Furthermore, NH_4_^+^ concentrations stood at 1.07 μg/m³ in PM_2.5_ and 1.00 μg/m³ in PM_1.0_, showing negligible differences. These data suggest that a majority of the water-soluble ions in PM_2.5_ are of PM_1.0_ dimension.

**Fig. 2:**
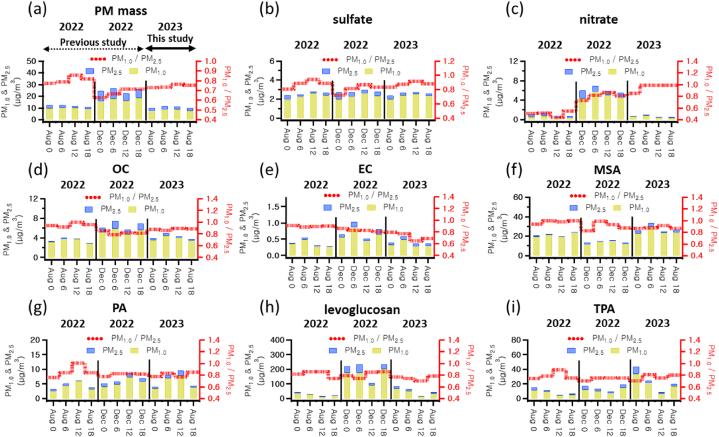
6-Hour Integrated Time-of-Day analysis in August and December 2022 and August 2023 for (a) PM Mass, (b) Sulfate, (c) Nitrate, (d) OC, (e) EC, (f) MSA, (g) PA, (h) Levoglucosan, and (i) TPA.

Fig. 2 illustrates the seasonal and diurnal variations in the concentration and composition of PM_2.5_ and PM_1.0_. Data from 2022 at the same location, using identical analytical methods, were included for comparison [16]. The concentrations of PM_2.5_ and PM_1.0_ did not exhibit significant variations during the summer observation periods of both years, and the hourly ratios of PM_2.5_ to PM_1.0_ were consistent as well. However, during the winter observation period, concentrations of PM_2.5_ and PM_1.0_ were notably higher than in the summer, with a greater PM_2.5_/PM_1.0_ ratio observed in winter. This escalation is likely due to enhanced combustion of fossil fuels for heating, elevating levels of WIOC and EC, alongside the seasonal stability of NO_3_^−^ at lower temperatures [16,[43], [44], [45]]. Notably, as depicted in Fig. 2(d) and (e), the ratios of OC and EC in PM_2.5_ and PM_1.0_ were elevated in winter relative to summer. Additionally, the concentration of levoglucosan, a marker for biomass burning, was higher in winter as presented in Fig. 2(h) [[46], [47], [48], [49]]. Furthermore, the concentration of NO_3_^−^ was greater in winter, as shown in Fig. 2(c).

In summary, the increase in PM during the winter observation period as compared to summer can be primarily attributed to combustion sources, yielding larger primary particles. Moreover, indicators of secondary organic carbon, such as PA, MSA, and TPA, have demonstrated a gradual rise during the summer observation periods. These components were predominantly detected in PM_1.0_, indicative of the formation of secondary particles. Specifically, PA concentrations increased from 12:00 to 18:00, suggesting photochemical reaction activities, while MSA peaked during peak commuting hours from 06:00 to 12:00 and 18:00 to 24:00, although its specific linkage to vehicular emissions demands further exploration [19,46,50]. TPA, another indicator of secondary organic carbon, demonstrated the highest concentrations from 00:00 to 06:00 during all observation periods, exhibiting more substantial increases compared to PA and MSA. This trend suggests a continuous rise in levels.

### 3.2 PM_2.5_ and PM_1.0_ human risk assessment and emission source estimation

A health risk assessment for PM_2.5_ and PM_1.0_ was performed, utilizing the concentrations of PAH components within these particles to calculate indices such as BaP_TEQ_, BaP_MEQ_, BaP_PEQ_, and HI (Table 2). Moreover, the assessment included QDTT-OP measurements for evaluating ROS, which are known to induce inflammatory responses in humans. Given the higher ambient concentration of PM_2.5_ compared to PM_1.0_, the focus was on risk per unit mass to enable an accurate comparison. BaP_TEQ_ and BaP_MEQ_, based on PAH concentrations, are calculated using TEF to represent its carcinogenicity and mutagenic potential, respectively [27,30]. BaP_PEQ_ relies on the PEF to gauge the carcinogenic potential of other PAH constituents relative to BaP. Additionally, HI quantifies non-carcinogenic risks associated with inhalation, dermal contact, and ingestion of other, less harmful PAHs like NaP, Acy, Ace, Flu, Phen, Ant, Flt, and Pyr. The QDTT-OP measurement is indicative of the rate of DTT depletion by ROS produced by particulate matter.

**Table 2:** Human risk index of PM_2.5_ and PM_1.0_ in study area.

Index	Unit	Time-of-Day in PM_2.5_					Time-of-Day in PM_1.0_					PM_1.0_/PM_2.5_
		**0**–**6**	**6**–**12**	**12**–**18**	**18**–**24**	**AVG**	**0**–**6**	**6**–**12**	**12**–**18**	**18**–**24**	**AVG**	
**BaP**_**TEQ**_	ng/mg	112	79	79	113	96 ± 19	156	109	118	140	131 ± 21	1.36
**BaP**_**TEQ**_	ng/mg	109	78	78	111	94 ± 19	152	107	117	137	128 ± 20	1.36
**BaP**_**MEQ**_	ng/mg	88	59	52	86	71 ± 18	120	83	81	108	98 ± 19	1.38
**BaP**_**PEQ**_	ng/mg	48	31	27	53	40 ± 13	62	46	44	61	53 ± 10	1.33
**HI**	ng/mg	0.46	0.41	0.43	0.44	0.44 ± 0.02	0.59	0.45	0.44	0.52	0.50 ± 0.07	1.14
**QDTT-OP**	nM/mg	34	32	26	32	31 ± 3	42	41	32	38	38 ± 4	1.23
**Overall average**												1.30

The health risk indices for BaP_TEQ_, BaP_MEQ_, BaP_PEQ_, HI, and QDTT-OP per unit mass of PM_2.5_ and PM_1.0_ are detailed in Table S1. Both the Nibet and LaGoy method, which calculates BaPTEQ using 16 PAHs, and the USEPA (1993) method, employing 7 PAHs, reveal that PM_1.0_ exhibits higher carcinogenicity than PM_2.5_. Specifically, BaPTEQ calculated by the Nibet and LaGoy method showed that PM_1.0_ had approximately 37 % higher carcinogenicity than PM_2.5_, while the USEPA (1993) method showed a 36 % higher value for PM_1.0_. Similarly, BaP_MEQ_, indicating mutagenic potential, was approximately 37 % higher for PM_1.0_ than for PM_2.5_, and BaP_PEQ_ was about 33 % higher for PM_1.0_. Thus, BaP_TEQ_, BaP_MEQ_, and BaP_PEQ_, all based on BaP equivalency factors, demonstrate that PM_1.0_ has at least a 33_1.0_ has at least 33 % greater carcinogenic and mutagenic potential than PM_2.5_. This augmented risk can be attributed to the particle size impact on the individual PAH components. The total concentration of PAHs was 3.00 ng/m³ for PM_2.5_ and 2.26 ng/m³ for PM_1.0_, with PM_2.5_ exhibiting roughly 33 % higher PAH concentrations. However, the determinations of BaP_TEQ_, BaP_MEQ_, and BaP_PEQ_ relied upon the levels of specific PAHs including BaA, Chry, BaP, DahA, IndP, BbF, and BkF. Except for BaP and BahA, the concentration variances between PM_2.5_ and PM_1.0_ for these PAHs were negligible. This suggests that highly carcinogenic and mutagenic PAHs, such as BaA, Chry, BaP, DahA, IndP, BbF, and BkF, predominate in the PM_1.0_ size fraction, underscoring the significance of managing PM_1.0_ to protect public health.

The average age-specific HI for non-carcinogenic risk was about 15 % higher for PM_1.0_ than for PM_2.5_. The HI index was notably 16 times greater for newborns than for adults, indicating significantly higher oral and dermal toxicity for newborns compared to adults. Contrarily, inhalation toxicity was over twice as significant for children than for adults, though newborns exhibited lower inhalation toxicity than adults. The detailed concentrations of non-carcinogenic substances such as NaP, Acy, Ace, Flu, Phen, Ant, Flt, and Pyr were investigated. Concentrations of NaP, Acy, Ace, and Flt approached zero in both PM_2.5_ and PM_1.0_, with the HI index primarily influenced by Flu, Phen, Ant, and Pyr. Specifically, the PM_1.0_/PM_2.5_ ratio for Flu, Phen, and Ant was around 0.9, suggesting that they were mostly found in PM_1.0_. Conversely, Pyr had a PM_1.0_/PM_2.5_ ratio of 0.3, indicating a higher concentration in PM_2.5_.

Furthermore, the ratio of C29αβ to C30αβ hopanes, induced by fossil fuel combustion, provides insights into the source: values below 1 imply traffic emissions, while values above 1 denote coal combustion sources [[51], [52], [53]]. In this analysis, the C29αβ/C30αβ ratios were 0.36 for PM_2.5_ and 1.20 for PM_1.0_, suggesting that while PM_2.5_ PAHs are mainly influenced by gasoline and diesel combustion, PM_1.0_ PAHs are predominantly from coal combustion.

The risk from ROS, assessed using the QDTT-OP method, was approximately 23 % greater for PM_1.0_ than for PM_2.5_. In summary, health risk indices such as BaP_TEQ_, BaP_MEQ_, BaP_PEQ_, HI, and QDTT-OP confirm that PM_1.0_ presents higher health risks than PM_2.5_ in this study.

### 3.3 Temporal analysis of health risk indices and combustion-derived levoglucosan, TPA, and PAHs in PM_1.0_

**Fig. 3:**
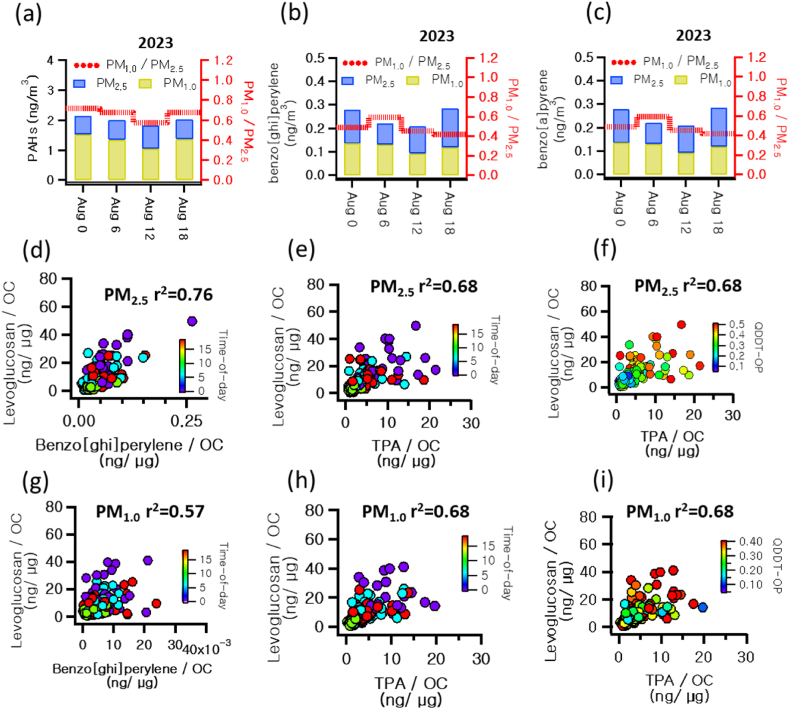
6-Hour Integrated Time-of-Day Analysis in August 2023 for (a) PAHs, (b) Benzo (ghi)perylene, (c) Benzo(a)pyrene, and scatter plots of (d,g) Levoglucosan/OC and Benzo (ghi)perylene/OC, (e,h) TPA/OC colored by Time-of-Day, and (f,i) QDTT-OP for PM_2.5_ and PM_1.0_, respectively.

To investigate the factors influencing the health risks associated with PM in the study area, we analyzed changes in health risk indices and component analysis over time. As demonstrated in Table 2, the health risks as measured by BaP_TEQ_, BaP_MEQ_, BaP_PEQ_, and HI were greatest during the hours of 00–06 and the next highest during 18–24. QDTT-OP also showed the highest health risk at 00–06, followed by 06–12. This suggests that the period from 00 to 06 is when particulate matter in the study area poses the greatest health risks. We consequently pinpointed the components that were elevated during the 00–06 window compared to other time periods. Fig. 2(h) and (i) highlighted levoglucosan and TPA, alongside PAH components depicted in Fig. 3.

**Fig. 4:**
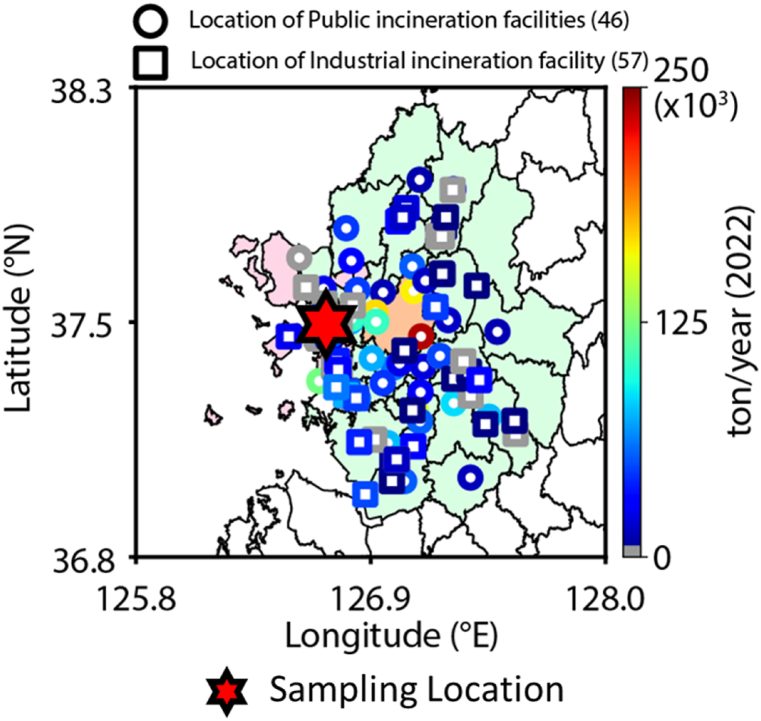
Number and locations of combustion facilities around the study area.

Approximately 70 % of the total incineration byproducts are TPA, stemming from open burning of PET [21]. TPA not only raises health concerns like cystitis but also acts as an indicator for PET combustion [20]. As evident in Fig. 2(i), TPA was primarily found in particulate matter during nighttime, suggesting its production from PET thermal decomposition, and implying that TPA in the study area results from combustion activities. Levoglucosan and TPA originate from the combustion of cellulose and PET respectively, while PAHs are produced from a wide array of combustion processes, including biomass burning and fossil fuel combustion. To identify common combustion activities influencing levoglucosan, PAHs, and TPA in the study area, we examined sources of local emissions. Fig. 4 illustrates that various combustion facilities, such as waste incinerators, are located around the study area. These facilities incinerate waste containing cellulose-based paper and PET, using coal as a fuel, potentially leading to emissions of levoglucosan, PAHs, and TPA. To further validate this, we studied the emission correlations of levoglucosan, PAHs, and TPA. Fig. 3 showcases that the correlation between levoglucosan/OC and BghiP/OC among PAHs at nighttime was notably high, with an r^2^ of 0.76, and the correlation between levoglucosan/OC and TPA/OC was also robust at r^2^ of 0.68. These findings imply that levoglucosan, PAHs, and TPA originate from shared combustion sources.

Moreover, as previously mentioned, the C29αβ/C30αβ ratio suggests that the PAHs in PM1.0 originate from coal combustion. Detailed examination shows that the daily average C29αβ/C30αβ ratio in PM_1.0_ was 1.20, but it was highest at 1.6 during 00–06, the period with the highest overall ratio. This suggests that PAHs in PM_1.0_ are significantly influenced by nighttime waste combustion using coal fuel. Additionally, Fig. 3 shows that during the nighttime, both levoglucosan/OC and TPA/OC concentrations increase, correlating with elevated QDTT-OP, which signifies a human health risk. In conclusion, levoglucosan, PAHs, and TPA in the study area originate from identical combustion sources, and the particulate matter they emit poses significant health risks, especially those components predominantly found in PM_1.0_ size particles.

## 4 Conclusion

This study evaluated the health risks and chemical compositions of PM_1.0_ and PM_2.5_ in Incheon, South Korea, highlighting the critical role of particle size in assessing public health impacts. Primary components detected in PM_1.0_ include levoglucosan and terephthalic acid (TPA), markers indicating combustion activities. The detection of these components in PM_1.0_ during nighttime suggests substantial contributions from combustion sources, such as biomass burning and plastic incineration. This research emphasized that PM_1.0_ carries a greater carcinogenic and mutagenic risk than PM_2.5_, as demonstrated by the elevated BaP_TEQ_, BaP_MEQ_, and BaP_PEQ_ values. PM_1.0_ showed 33–37 % higher carcinogenic and mutagenic risks than PM_2.5_, despite lower total PAH levels, indicating that highly toxic PAHs are more prevalent in PM_1.0_. Non-carcinogenic risks were 15 % higher for PM_1.0_. The C29αβ/C30αβ hopane ratio suggested traffic emissions for PM_2.5_ and coal combustion for PM_1.0_. QDTT-OP showed 23 % greater ROS-related risks for PM_1.0_. The overall average risk index for PM_1.0_ is 1.30 times higher than PM_2.5_, implying that, on average, PM_1.0_ poses a 30 % higher health risk across the measured indices compared to PM_2.5_. These results underscore the critical need for improved management of PM_1.0_ to mitigate public health risks. The correlation between levoglucosan, PAHs, and TPA supports the theory that these substances originate from common combustion sources, notably waste incinerators. Moreover, analysis of the C29αβ/C30αβ hopane ratio indicated a significant contribution of waste combustion to PAHs in PM_1.0_, particularly at night. The increased health risks from QDTT-OP confirm that PM_1.0_ poses considerable health dangers due to its chemical composition and sources of emissions. In conclusion, this study demonstrates that PM_1.0_, primarily formed by secondary aerosol processes and combustion activities, poses significant health risks. Effective management of emission sources, especially those related to combustion, is crucial for reducing the health impacts of particulate matter, focusing on mitigating risks associated with PM_1.0_. These findings lay a solid foundation for future research and public health policies designed to enhance air quality and protect human health.

## CRediT authorship contribution statement

**Myoungki Song:** Writing – original draft, Methodology, Conceptualization. **Seoyeong Choe:** Investigation, Data curation. **Sea-Ho Oh:** Formal analysis, Data curation. **Minyoung Sung:** Validation, Data curation. **Ji Yun Jung:** Investigation, Formal analysis. **Jinsoo Choi:** Formal analysis, Data curation. **Joonyoung Ahn:** Investigation, Formal analysis. **Jungmin Park:** Validation, Investigation. **Myungsoo Yoo:** Methodology, Formal analysis. **Jinsoo Park:** Writing – review & editing, Investigation. **Min-Suk Bae:** Writing – review & editing, Supervision, Conceptualization.

## Data availability

Data from this study are available from the corresponding author upon reasonable request.

## Declaration of Competing Interest

The authors declare that they have no known competing financial interests or personal relationships that could have appeared to influence the work reported in this paper.
