# Supplementary material for: Temporal variations in health risk indices and combustion-derived components of PM1.0: Focus on terephthalate and levoglucosan
Source: Heliyon. 2024 Nov 9;10(22):e40052. doi: 10.1016/j.heliyon.2024.e40052 (PMC11600074; doi:10.1016/j.heliyon.2024.e40052)
Supplement: Multimedia component 1 [file mmc1.docx]

**Temporal Variations in Health Risk Indices and Combustion-Derived Components of PM_1.0_: Focus on Terephthalate and Levoglucosan**

Myoungki Song ^a^, Seoyeong Choe, Sea-Ho Oh ^a^, Minyoung Sung ^b^, Ji Yun Jung ^b^, Jinsoo Choi ^b^, Joonyoung Ahn ^b^, Jungmin Park ^b^, Myungsoo Yoo ^c^, Jinsoo Park ^b,1,^**, Min-Suk Bae ^a,1,^*

^a^ Department of Environmental Engineering, Mokpo National University, Muan, Republic of Korea

^b^ Climate and Air Quality Research Department, Air Quality Research Division, National Institute of Environmental Research, Incheon, Republic of Korea

^c^ Climate and Air Quality Research Department, National Institute of Environmental Research, Incheon, Republic of Korea

^⁎^ Corresponding author.

^⁎⁎^ Corresponding author.

^1^ Jinsoo Park and Min-Suk Bae contribute equally to this work and should be considered as co-corresponding authors.

**Assessment of Carcinogenic and Non-Carcinogenic Risks of Polycyclic Aromatic Hydrocarbons (PAHs) in Urban Air Using BaP_TEQ_, BaP_MEQ_, and BaP_PEQ_ Models**

The formulas for calculating BaP_TEQ_, BaP_MEQ_, and BaP_PEQ_ are presented in Equations 1 to 3. The PEFs used in these calculations are shown in Table 1.

${BaP}_{TEQ}= \Sigma(C_{i}\times{BaP}_{TEF})$ eq. (1)

${BaP}_{MEQ}= \Sigma(C_{i}\times{BaP}_{MEF})$ eq. (2)

${BaP}_{PEQ}= \Sigma(C_{i}\times{BaP}_{PEF})$ eq. (3)

The non-carcinogenic risk of PAHs was assessed using non-carcinogenic substances contained in the particles, such as NaP, Acy, Ace, Flu, Phen, Ant, Flt, and Pyr. Specifically, the non-carcinogenic risk was calculated for oral, dermal, and inhalation exposures across different age groups, ultimately expressing the risk index (HI) for each age group. The non-carcinogenic risk of PAHs was calculated using Equations 4 to 7.

${CDI}_{ingestion}= \frac{C_{UCL}\times IngR\times EF\times ED}{BW\times AT}\times{10}^{-6}$ eq. (4)

${CDI}_{inhalation}= \frac{C_{UCL}\times InhR\times EF\times ED}{PEF\times BW\times AT}$ eq. (5)

${CDI}_{dermal}= \frac{C_{UCL}\times SA\times AF\times ABS\times EF\times ED}{BW\times AT}\times{10}^{-6}$ eq. (6)

$\mathrm{HI}= {CDI}_{ingestion}+{CDI}_{inhalation}+{CDI}_{dermal}$ eq. (7)

Here, CDI_ingestion_, CDI_inhalation_, and CDI_dermal_ refer to the non-carcinogenic risks associated with ingestion, inhalation, and dermal exposure, respectively. C_UCL_ represents the 95% upper confidence level (UCL) concentrations of NaP, Ace, Acy, Flu, Phen, Ant, Flt, and Pyr. EF stands for the exposure frequency, set at 350 days/year. ED denotes the exposure duration, BW is the body weight by age group, and AT is the averaging time for non-carcinogenic risk, calculated as ED × 365 days. PEF is the particle emission factor, set at 1.36 × 10^9^ m^3^/kg. SA represents the skin surface area, AF is the skin adherence factor, and ABS is the dermal absorption factor, set at 0.13. The age-specific indicators for each index are presented in Table S2.

**Table S1.** PAHs toxic equivalent factors (TEFs), mutagenic equivalents factors (MEFs) and potency equivalency factors (PEFs) with PAHs mean concentrations.

| Compounds | TEF |  | MEF | PEF |
| --- | --- | --- | --- | --- |
|  | Nidbet and LaGoy (1992) | USEPA (1993) | Durant et al.  (1999) | OEHHA (1994) |
| Ace | 0.001 |  |  |  |
| Acy | 0.001 |  |  |  |
| Ant | 0.01 |  |  |  |
| BaA | 0.1 | 0.1 | 0.082 | 0.1 |
| BaP | 1 | 1 | 1 | 1 |
| BbF | 0.1 | 0.1 | 0.25 | 0.1 |
| BkF | 0.1 | 0.01 | 0.11 | 0.1 |
| BghiP | 0.01 |  | 0.19 |  |
| Chry | 0.01 | 0.001 | 0.017 | 0.001 |
| DahA | 1 | 1 | 0.29 |  |
| Flt | 0.001 |  |  |  |
| Flu | 0.001 |  |  |  |
| IndP | 0.1 | 0.1 | 0.31 | 0.1 |
| NaP | 0.001 |  |  |  |
| Phen | 0.001 |  |  |  |
| Pyr | 0.001 |  |  |  |

**Table S2.** Variables for estimation of human health risk assessment

| Variables | Units | Infant^3)^ | Toddler^3)^ | Child^3)^ | Teen^3)^ | Adult^3)^ |
| --- | --- | --- | --- | --- | --- | --- |
| Age | years | 0-0.5 | 0.6-5 | 6-12 | 13-20 | 21-65 |
| Exposure duration (ED) | years | 0.5 | 4.5 | 7.0 | 8.0 | 34.5 |
| Body weight (BW) | kg | 8.2 | 16.5 | 32.9 | 59.7 | 65.0 |
| Ingestion rate (IngR) | mg/day | 200 | 200 | 200 | 100 | 100 |
| Skin adherence factor | mg/cm^2^ | 0.2 | 0.2 | 0.2 | 0.07 | 0.07 |
| Skin surface area | m^2^ | 203 | 344 | 586 | 908 | 1030 |
| Inhalation rate (InhR) | m^3^/day | 2.0 | 5.0 | 12.0 | 21.0 | 50.0 |
| 1) USEPA, 2012  2) Durant, 1996  3) Rout et al., 2013 | | | | | | |
